# DNA barcoding of coral reef fishes from Chuuk State, Micronesia

**DOI:** 10.1080/23802359.2020.1831981

**Published:** 2020-11-11

**Authors:** Jae Ho Choi, Da Geum Jeong, Ji Na Oh, Sung Kim, Youn Ho Lee, Young UngChoi, Jung Goo Myoung, Choong Gon Kim

**Affiliations:** a Department of Convergence Study on the Ocean Science and Technology Ocean Science and Technology School, Korea Maritime and Ocean University, Busan, Korea; b Marine Ecosystem Research Center, Korea Institute of Ocean Science and Technology, Busan, Korea; c Marine Bio-Resources Research Unit, Korea Institute of Ocean Science and Technology, Busan, Korea

**Keywords:** Coral reef fish, mitochondrial DNA COI, DNA barcode, identification, Chuuk State, Micronesia

## Abstract

The fish diversity of Chuuk Micronesia is currently under threat due to rapid changes in the coral reef ecosystem. Thus, accurate fish identification using DNA barcodes is fundamental for exploring species biodiversity and resource protection. In this study, we analyzed 162 fish mitochondrial DNA cytochrome *c* oxidase I (COI) barcodes from Chuuk Micronesia. Consequently, we identified 95 species from 53 genera in 26 families and seven orders. The average Kimura 2-parameter genetic distances within species, genera, families, and orders were calculated as 0.17%, 11.78%, 15.63%, and 21.90%, respectively. Also, we have utilized DNA barcodes to perform genetic divergence and phylogenetic analysis of families recognized as dominant groups in Chuuk State. Our findings confirm that DNA barcodes using COI are an effective approach in identifying coral reef fish species. We anticipate that the results of this study will provide baseline data for the protection of coral reef fish biodiversity at Chuuk Micronesia.

## Introduction

Micronesia, which is located in the Western Pacific Ocean, consists of four states (Yap, Chuuk, Pohnpei, and Kosrae) that collectively have a coral reef area exceeding 6000 km^2^ (Andréfouët et al. 2006). As growth and spawning grounds for a wide range of marine organisms, coral reefs are often characterized by their high biodiversity (Reaka-Kudla 1997). The reefs of Micronesia have served as a habitat for many species of corals, fishes, and invertebrates. Chuuk State consists of 18 major volcanic islands, many smaller and uninhabited islands, and a diversity of tropical marine reefs, ranging in size from 0.4 to 4.6 km^2^. Recently, population expansion, economic growth, and indiscriminate fishing have threatened the biodiversity of the region (Edward 2002). Further, global climate change is causing ocean acidification, rising sea levels, and rising water temperatures, changes that have been considered detrimental to the coral reef ecosystems and thus creating a crisis of marine biodiversity (Hoegh-Guldberg et al. 2007; Baker et al. 2008; Thompson and Van Woesik 2009).

Effective conservation and management of fish biodiversity require reliable baseline estimates of fish species diversity based on accurate species identification. Identification of fish species is traditionally based on morphology (Dayrat 2005; Triantafyllidis et al. 2011). However, morphological identification requires considerable expertise, given that the morphology of fish varies and often changes concomitantly with developmental stage (Leis and Carson-Ewart 2000; Wang et al. 2018). These issues can be addressed by DNA barcoding, which is based on pattern analysis of standardized gene regions. This approach has been identified to be more reliable for species identification (Hebert et al. 2003; Hebert and Gregory 2005). A 655-bp fragment of the mitochondrial COI gene is widely used for species-level identifications. Mitochondrial DNA shows a high mutation rate and large copy numbers. Organisms with small effective population sizes often provide genomes that are useful for analyses of evolutionary patterns and processes (Brown et al. 1979; Birky et al. 1989). Numerous previous studies around the world, including studies in Taiwan (Bingpeng et al. 2018), Pacific Canada (Steinke et al. 2009), Australia (Ward et al. 2005), the Philippines (Abdulmalik-Labe and Quilang 2019), China (Wang et al. 2018), India (Lakra et al. 2011), Turkey (Keskin and Atar 2013), and Japan (Zhang and Hanner 2011), have demonstrated the utility of COI barcodes in fish species identification.

We used mitochondrial DNA COI barcodes to identify some coral reef fish species from Chuuk State, Micronesia. These species can be difficult to identify by morphological identification.

## Materials and methods

### Sample collection

The research area is along the northeastern coast of Weno Island in Chuuk State (7°27′N, 151°51′E), where coral reefs are well developed. Fishes were collected by diving and netting or were purchased from a local market in 2006, 2007, 2008, and 2011.

### DNA isolation

Genomic DNA was extracted from tissue pieces using a Qiagen DNeasy Blood & Tissue Kits (QIAGEN, Valencia, CA, USA), following the manufacturer’s protocol. All gDNAs extracted from whole samples were stored at −20 °C at the Marine Ecosystem Research Center, Korea Institute of Ocean Science and Technology, Busan, Korea. The quality and quantity of extracted DNA were measured using a NanoDrop^®^ ND-1000 spectrophotometer (NanoDrop Technologies, Wilmington, USA).

### Amplification and sequencing

PCR amplification was performed using combinations of primers for fish 655-bp COI barcoding region (Ward et al. 2005). Thermal amplification reactions were performed in 25 μL reaction mixtures, which contained 1× PCR buffer, 2 mM MgCl_2_, 10 pmol of each primer, 0.25 mM of each dNTP, 0.25 U of Taq polymerase, and 100 ng of DNA template. The thermocycling program consisted of an initial step of 94 °C for 1 min; followed by 35 cycles of 94 °C for 30 s, 50 °C for 40 s, and 72 °C for 1 min; a final extension at 72 °C for 10 min; and a final hold at 4 °C. PCR products were then checked using 2% agarose gel electrophoresis. PCR products were purified using a QIAquick PCR Purification Kit (QIAGEN, Valencia, CA, USA), following the manufacturer’s protocol. Sequencing reactions were performed in an MJ Research PTC-225 Peltier Thermal Cycler using ABI PRISM BigDye™ Terminator Cycle Sequencing Kits with AmpliTaq DNA polymerase (FS enzyme) (Applied Biosystems), following the protocols provided by the manufacturer.

### Sequence analysis

All sequences were aligned and integrated using MEGA X (Kumar et al. 2018). Obtained sequences were then compared with sequences from NCBI GenBank databases. Samples with similarity indices greater than 97% compared with available database sequences were considered to be the same species. Nucleotide composition, transition(si)/transversion(sv) pair ratios, and K2P genetic distances, including intra- and interspecific divergences, were calculated using MEGA X. Neighbor-joining (NJ) phylogenetic tree (Saitou and Nei 1987) was constructed based on K2P genetic distance using MEGA X with bootstrap tests of 1000 replications were generated to verify the robustness of the tree. The K2P can be rapidly calculated, which in turn can provide consistent results for many species that show required differences between intra- and interspecies variability (Kimura 1980; Shen et al. 2016). The K2P model is commonly used in DNA barcoding (Zhang and Hanner 2011; Keskin and Atar 2013; Bingpeng et al. 2018; Wang et al. 2018).

## Results and discussions

Analysis of 162 COI DNA barcodes was able to identify 95 species, 53 genera, 26 families, and seven orders (Anguilliformes, Beloniformes, Beryciformes, Mugiliformes, Ophidiiformes, Perciformes, and Tetraodontiformes) among fishes from Chuuk State. We then obtained the NCBI accession numbers for all the specimens (Table 1). The COI barcode used in the analyses comprised 655 nucleotide base pairs per taxon, and no contamination, insertions, deletions, or stop codons were determined in any obtained sequence. Average K2P genetic distances within species, genera, families, and orders were determined to be 0.17%, 11.78%, 15.63%, and 21.90%, respectively. The average interspecific genetic distance increased concomitant with an increase in genetic variation at progressively higher taxonomic levels. DNA barcoding efficiency is then verified by intraspecific and interspecific distances (Lievens et al. 2001). Average intraspecific genetic distance is 0.3% in BOLD (Barcode of Life Data System) fish databases, and congeneric distance is at least 30-fold higher than conspecific distances (Zhang and Hanner 2011). Intraspecific distance and congeneric distance were determined to be 69-fold higher than conspecific distance in the current study. Our study confirmed that DNA barcodes are useful in identifying coral reef fish species. Moreover, we found that intraspecific genetic distances determined in this present study are less than the previously reported distances; in contrast, interspecific genetic distance was found to be greater.

**Table 1.** List of species analyzed for DNA barcodes and sequence information.

Order	Family	Genus/Species	GenBank accession no.	Voucher ID	*N*	Reference accession no.	Similarity (%)
Perciformes	Acanthuridae	*Acanthurus lineatus*	MN733529	CKF003	1	HM034183	100
		*Acanthurus nigricauda*	MN733530, MN733650	CKF004, CKF121	2	HM034188	100
		*Acanthurus triostegus*	MN733531, MN733532	CKF005, CKF006	2	JQ349668	100
		*Ctenochaetus striatus*	MN733528, MN733569, MN733570	CKF002, CKF042, CKF043	3	MK658679	99
		*Naso brevirostris*	MN733610, MN733665	CKF082, CKF134	2	KF930171	100
		*Naso lituratus*	MN733611, MN733612	CKF083, CKF084	2	HM034244	100
		*Naso unicornis*	MN733613, MN733614, MN733615	CKF085, CKF086, CKF087	3	KF714984	99
		*Naso vlamingii*	MN733616	CKF088	1	HQ564379	100
		*Zebrasoma velifer*	MN733649	CKF120	1	MK657444	100
	Ambassidae	*Ambassis miops*	MN733533, MN733678, MN733702	CKF007, CKF146, CKF160	3	HQ654651	99
	Apogonidae	*Cheilodipterus quinquelineatus*	MN733703	CKF161	1	KP194469	99
		*Fibramia lateralis*	MN733537, MN733538	CKF010, CKF011	2	KP194856	99
		*Sphaeramia orbicularis*	MN733639, MN733640, MN733641	CKF111, CKF112, CKF113	3	AP018927	100
		*Fibramia thermalis*	MN733539	CKF012	1	AB890041	99
	Blenniidae	*Blenniella paula*	MN733593	CKF066	1	MK658217	100
	Caesionidae	*Caesio caerulaurea*	MN733670	CKF138	1	KF009569	99
	Carangidae	*Carangoides plagiotaenia*	MN733651	CKF122	1	KC970456	100
		*Caranx melampygus*	MN733542	CKF015	1	KC970375	100
		*Selar boops*	MN733673	CKF141	1	KF009659	100
	Chaetodontidae	*Chaetodon ephippium*	MN733546, MN733547, MN733548 MN733549, MN733550, MN733551 MN733552, MN733553, MN733554 MN733555, MN733556, MN733557	CKF019, CKF020, CKF021 CKF022, CKF023, CKF024 CKF025, CKF026, CKF027 CKF028, CKF029, CKF030	12	JF434773	100
		*Chaetodon lunulatus*	MN733558	CKF031	1	KJ967960	100
		*Chaetodon ornatissimus*	MN733559	CKF032	1	JF434807	99
		*Chaetodon ulietensis*	MN733560	CKF033	1	FJ583101	99
	Gobiidae	*Amblygobius phalaena*	MN733700	CKF158	1	AF391369	99
		*Asterropteryx ensifera*	MN733541, MN733699, MN733679	CKF014, CKF157, CKF147	3	JX483981	100
	Kyphosidae	*Kyphosus cinerascens*	MN733594, MN733689	CKF067, CKF153	2	JQ350079	100
	Labridae	*Cheilinus chlorourus*	MN733562	CKF035	1	KF714912	99
		*Cheilinus trilobatus*	MN733561	CKF034	1	KF009582	100
		*Coris batuensis*	MN733568	CKF041	1	KP194597	100
		*Halichoeres margaritaceus*	MN733590	CKF063	1	JQ839484	99
		*Halichoeres marginatus*	MN733591	CKF064	1	AY850781	100
		*Halichoeres melanurus*	MN733589	CKF062	1	KP194607	98
		*Halichoeres trimaculatus*	MN733592	CKF065	1	KP194873	100
		*Oxycheilinus celebicus*	MN733617	CKF089	1	HQ564433	99
		*Oxycheilinus digramma*	MN733618, MN733619	CKF090, CKF091	2	KP194504	100
		*Stethojulis bandanensis*	MN733643	CKF115	1	KP194849	100
	Lethrinidae	*Lethrinus erythropterus*	MN733598, MN733660	CKF071, CKF130	2	HM902431	100
		*Lethrinus obsoletus*	MN733595, MN733596	CKF068, CKF068	2	AP009165	99
		*Lethrinus olivaceus*	MN733597	CKF070	1	KJ968135	99
		*Lethrinus xanthochilus*	MN733659, MN733661	CKF129, CKF131	2	KP194924	100
		*Monotaxis grandoculis*	MN733604, MN733605	CKF077, CKF078	2	AP009166	99
		*Monotaxis heterodon*	MN733606, MN733663	CKF079, CKF133	2	MK657454	100
	Lutjanidae	*Lutjanus fulvus*	MN733599, MN733600	CKF072, CKF073	2	KF009613	99
		*Lutjanus decussatus*	MN733601	CKF074	1	MN870144	100
		*Macolor macularis*	MN733602, MN733686	CKF075, CKF150	2	EF609403	100
		*Macolor niger*	MN733662	CKF132	1	KF489639	100
	Monodactylidae	*Monodactylus argenteus*	MN733603	CKF076	1	AP009169	100
	Mullidae	*Mulloidichthys flavolineatus*	MN733607, MN733608	CKF080, CKF081	2	MN870473	100
		*Parupeneus barberinus*	MN733620	CKF092	1	AP018401	100
		*Parupeneus cyclostomus*	MN733667	CKF136	1	MK658446	100
		*Parupeneus insularis*	MN733666	CKF135	1	JQ431985	99
		*Parupeneus multifasciatus*	MN733621	CKF093	1	AP012314	99
	Pomacentridae	*Abudefduf vaigiensis*	MN733527	CKF001	1	AP006016	99
		*Amblyglyphidodon curacao*	MN733535, MN733536	CKF008, CKF009	2	KF929588	100
		*Chromis viridis*	MN733676	CKF144	1	MT199208	100
		*Chrysiptera glauca*	MN733625, MN733692	CKF097, CKF154	2	JQ707144	98
		*Neopomacentrus azysron*	MN733626	CKF098	1	KP194962	100
	Scaridae	*Cetoscarus bicolor*	MN733544, MN733545	CKF017, CKF018	2	AY662758	99
		*Chlorurus bleekeri*	MN733563, MN733655	CKF036, CKF125	2	MN870261	100
		*Chlorurus frontalis*	MN733653	CKF124	1	JQ431617	100
		*Chlorurus sordidus*	MN733565, MN733566, MN733567	CKF038, CKF039, CKF040	3	AP006567	99
		*Chlorurus microrhinos*	MN733564	CKF037	1	JN313047	99
		*Scarus chameleon*	MN733628, MN733629	CKF100, CKF101	2	FJ237915	100
		*Scarus ghobban*	MN733656	CKF126	1	FJ449707	99
		*Scarus niger*	MN733672	CKF140	1	JQ432105	99
		*Scarus oviceps*	MN733631	CKF103	1	JQ432106	100
		*Scarus psittacus*	MN733630, MN733632	CKF102, CKF104	2	MK658527	100
		*Scarus rubroviolaceus*	MN733633	CKF105	1	FJ227899	99
		*Scarus schlegeli*	MN733671	CKF139	1	JQ432114	100
		*Hipposcarus longiceps*	MN733695	CKF155	1	KF929973	100
	Scombridae	*Thunnus albacares*	MN733644, MN733645	CKF116, CKF117	2	KP259550	99
	Serranidae	*Aethaloperca rogaa*	MN733698	CKF156	1	KC593376	100
		*Cephalopholis argus*	MN733543	CKF016	1	MF185407	100
		*Epinephelus polyphekadion*	MN733585, MN733586, MN733571 MN733572, MN733573, MN733574 MN733575, MN733576, MN733577 MN733578, MN733579, MN733580 MN733581, MN733582	CKF058, CKF059, CKF044 CKF045, CKF046, CKF047 CKF048, CKF049, CKF050 CKF051, CKF052, CKF053 CKF054, CKF055	14	MH707787	100
		*Epinephelus howlandi*	MN733583, MN733657	CKF056, CKF127	2	MH707757	100
		*Epinephelus merra*	MN733584	CKF057	1	KC970471	99
		*Epinephelus spilotoceps*	MN733658	CKF128	1	MH707800	100
		*Plectropomus areolatus*	MN733668	CKF137	1	KC262636	100
		*Plectropomus laevis*	MN733622	CKF094	1	KP194704	100
		*Plectropomus oligacanthus*	MN733623, MN733624	CKF095, CKF096	2	HM422409	99
		*Variola louti*	MN733647, MN733648	CKF118, CKF119	2	KC593369	100
	Siganidae	*Siganus argenteus*	MN733675	CKF143	1	MN870479	100
		*Siganus guttatus*	MN733635, MN733674	CKF107, CKF142	2	KJ420577	99
		*Siganus virgatus*	MN733634	CKF106	1	KF715023	99
		*Siganus stellatus*	MN733636, MN733637	CKF108, CKF109	2	KT997948	100
		*Siganus vulpinus*	MN733638	CKF110	1	FJ584115	100
	Sphyraenidae	*Sphyraena jello*	MN733642	CKF114	1	HM422420	99
		*Sphyraena qenie*	MN733677	CKF145	1	MK657164	100
Tetraodontiformes	Tetraodontidae	*Arothron manilensis*	MN733540	CKF013	1	AP011929	99
Beloniformes	Zenarchopteridae	*Zenarchopterus dispar*	MN733682, MN733704	CKF148, CKF162	2	KP194857	99
Ophidiiformes	Carapidae	*Carapus mourlani*	MN733652	CKF123	1	KU681392	100
Beryciformes	Holocentridae	*Sargocentron spiniferum*	MN733627	CKF099	1	KP194463	100
		*Neoniphon sammara*	MN733685, MN733701	CKF149, CKF159	2	MG816708	100
Mugiliformes	Mugilidae	*Moolgarda engeli*	MN733687	CKF151	1	MG816710	100
Anguilliformes	Muraenidae	*Gymnothorax pictus*	MN733587, MN733588, MN733688	CKF060, CKF061, CKF152	3	KP194043	99

All COI reference databases were derived from GenBank. (*N*: Number of individuals).

Average nucleotide composition of the 162 DNA barcodes was T = 29.08%, C = 28.39%, A = 24.18%, and G = 18.35%. The average GC and AT contents were 46.74% and 53.26%, respectively. The highest (52.76%) and lowest (38.51%) GC values were detected in COI barcodes of *Fibramia thermalis* and *Zenarchopterus dispar*. Further, the average ratio (si/sv) of all specimens has been determined to be 1.38. Divergence time among specimens was analyzed in terms of transition(si)/transversion(sv) ratio and genetic distance. The former is considered a general property of DNA sequence evolution. This ratio provides a reliable estimate of sequence distance and can be further used in phylogeny reconstruction. A high si/sv ratio is indicative of a small genetic distance, and vice versa (Yang and Yoder 1999). We were able to analyze the divergence times among families, for example, Acanthuridae, Labridae, Scaridae, and Serranidae, which are dominant in Chuuk Micronesia using DNA barcodes of the fish collected in this study. Average si/sv ratios for these families were 2.10, 1.56, 3.5, and 1.8, respectively. Further, the mean genetic distances among species within families were 16.08%, 20.25%, 11.15%, and 18.80%, respectively. Scaridae family displays the highest si/sv ratio (3.5) and the lowest genetic distance among species within families (11.15%). Scaridae appears to be a recently diverged group and is youngest among dominant families in Chuuk State, Micronesia. Moreover, compared with other families with similar divergence times, we collected a larger number of species in the Scaridae. It is predicted that Scaridae is well adapted to the rich coral reef found at Chuuk State. In contrast, the Labridae family has showed the highest genetic distance (20.25%) and lowest si/sv ratio (1.74) among major groups. This result may reflect an early divergence of species in the Labridae.

**Figure 1.**
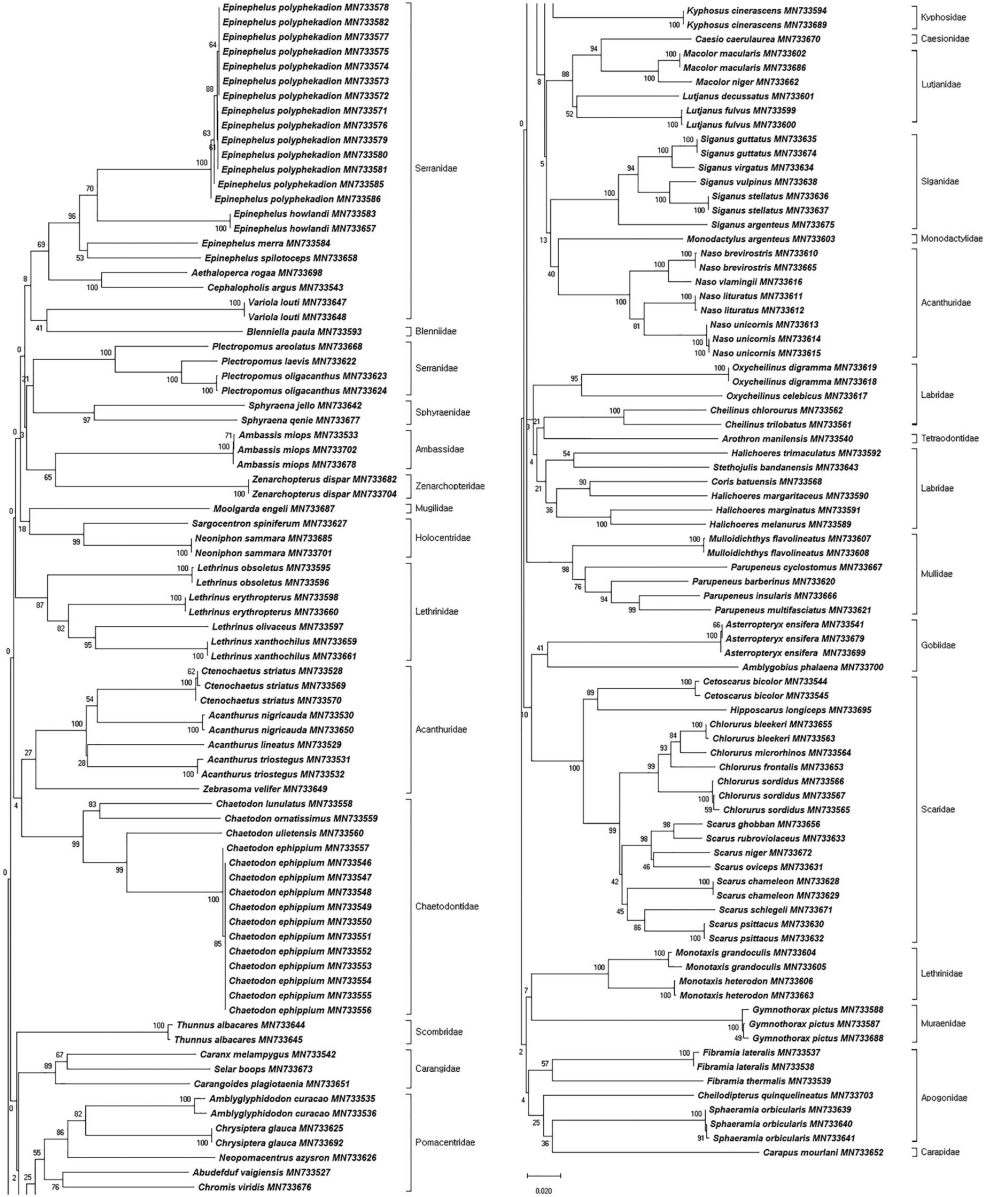
Neighbor-joining (NJ) tree of 162 COI barcodes using K2P distances.

The NJ tree from 162 specimens was constructed based on K2P distances (Figure 1). We used this tree to confirm that all species were clustered monophyletic. Thus, DNA barcode analysis is effective in identifying species known to be similar based on morphological observation. Confamilial species are then classified and grouped as independent clades in general phylogenetic analysis. However, some families in this study (Acanthuridae, Serranidae, and Labridae) were not grouped together. Mitochondrial DNA evolves faster than nuclear DNA and is characterized by larger numbers of variable and informative sites. Rapid substitution rates of mitochondrial DNA also make it useful for analyses at species and genus levels. However, deeper branching may then reduce saturation, which can result in homoplasy, as the phylogenetic signal has been reduced (Caterino et al. 2001; Rubinoff and Sperling 2002; Rubinoff and Holland 2005). A previous study (Ward et al. 2005) suggests that phylogenetic analysis using single mitochondrial DNA is suitable for simpler studies, not for deep phylogenetic analysis. Therefore, we confirmed that mitochondrial DNA COI barcodes are effective for identification of coral reef fish species and analysis of phylogenetic relationships at the species and genus level.

This study, to the best of our knowledge, is the first in which mitochondrial DNA COI barcodes have been used in analyzing coral reef fishes in Chuuk, Micronesia. We identified 95 species, 53 genera, 26 families, and seven orders based on DNA barcoding of 162 fish specimens. Furthermore, we have analyzed divergence time and phylogenetic relationships of fish families that are dominant groups in Chuuk State. Our results confirm that the mitochondrial COI DNA barcodes are an effective tool for the identification of coral reef fish. We predict that similar analyses using larger sample sizes would yield more accurate results given the high marine biodiversity of the study area. We thus anticipate that DNA barcode information obtained in this study will provide baseline data for the protection of coral reef fish biodiversity in Chuuk State, Micronesia.

## Data Availability

The data that support the findings of this study are openly available in NCBI at https://www.ncbi.nlm.nih.gov/, all reference numbers in Table 1.
